# Development and psychometric validation of the hospitalized patients’ expectations for treatment scale-clinician version

**DOI:** 10.3389/fpsyt.2023.1325013

**Published:** 2024-01-12

**Authors:** Bindong Dai, Chunfeng Xiao, Yufei Wang, Tao Li, Yanping Duan, Yinan Jiang, Lili Shi, Xia Hong, Wenqi Geng, Jiaojiao Hu, Jinya Cao, Jing Wei

**Affiliations:** ^1^Department of Psychological Medicine, Peking Union Medical College Hospital, Chinese Academy of Medical Sciences and Peking Union Medical College, Beijing, China; ^2^4+4 Medical Doctor Program, Chinese Academy of Medical Sciences and Peking Union Medical College, Beijing, China

**Keywords:** clinician, general hospital, treatment expectation, doctor-patient relationship, patient safety

## Abstract

**Objective:**

Patient safety management systems in general hospitals require a comprehensive tool for assessing the expectations of inpatients across different wards. This study aimed to develop and psychometrically validate a new scale, the hospitalized patients’ expectations for treatment scale-clinician version (HOPE-C), to meet this requirement.

**Methods:**

We interviewed 35 experts and 10 inpatients while developing the HOPE-C scale. The scale was initially designed with three dimensions: clinicians’ expectations regarding doctor-patient communication, clinicians’ expectations regarding treatment outcome, and clinicians’ expectations regarding disease management. We recruited 200 inpatients from a general hospital in China. At the same time, 51 clinicians were assigned to the enrolled patients who completed the HOPE-C to examine the reliability, validity, and psychometric characteristics of the questionnaire. We applied item analysis, assessed construct validity, evaluated internal consistency, and conducted a test-retest reliability analysis over 7 days.

**Results:**

Both exploratory and confirmatory analyses supported a 2-dimensional structure, comprising doctor-patient communication expectations and treatment outcome expectations, with favorable model fit parameters (root mean square residual [RMR] = 0.042, root mean square error of approximation [RMSEA] = 0.049, comparative fit index [CFI] = 0.989, Tucker-Lewis index [TLI] = 0.984). Item analysis demonstrated appropriate item design (*r* = 0.744–0.961). The scale exhibited strong internal consistency, with Cronbach’s α values of 0.884, 0.816, and 0.840 for the overall scale, the doctor-patient communication expectation subscale, and the treatment outcome expectation subscale, respectively. The 7-day test-retest reliability was 0.996 (*p* < 0.001).

**Conclusion:**

Our findings suggest that the HOPE-C is a reliable and valid assessment tool for measuring the expectations of inpatients in general hospitals. It effectively identifies patients’ expectations concerning doctor-patient communication and treatment outcomes.

## 1 Introduction

In healthcare, understanding physicians’ expectations for treatment is paramount for optimizing patient care. Since physicians play a pivotal role in patient treatment, their expectations can considerably impact healthcare delivery, patient outcomes, and the overall quality of healthcare services. With a growing emphasis on patient-centered care and shared decision-making, understanding physicians’ expectations for treatment has become a central concern (1). First, physicians are responsible for diagnosing and treating medical conditions and establishing and maintaining the patient-physician relationship, serving as the foundation of patient-centered care (2). Second, as trusted healthcare providers, physicians bring their own expectations, beliefs, and preferences into their clinical practice, influencing the clinical decisions they make for their patients (3). Furthermore, trust and communication between physicians and patients are pivotal in achieving optimal health outcomes, patient satisfaction, and adherence to treatment plans (4).

The importance of understanding physicians’ expectations lies in its potential to improve healthcare outcomes. A deeper comprehension of what physicians expect can help healthcare institutions and policymakers design strategies and interventions to align these expectations with patient-centered care goals. It can also enhance medical education and training by tailoring curricula to address areas where expectations differ from patient needs and preferences.

Physicians’ expectations are a notable factor influencing clinical practice, which might affect patient outcomes. Previous studies demonstrated a gap in patient-physician communication regarding expectations of patient treatment outcomes (5). In cancer survivorship care, patients and physicians have discordant expectations (6, 7). Moreover, an enhanced agreement between patients’ preferences and physicians’ expectations can improve communication and patients’ satisfaction with treatment (8–10). Shared decision-making (SDM) with the incorporation of patient-reported outcomes can promote patient adherence and satisfaction (11). The concordance of treatment expectations is the key factor in SDM, potentially helping manage patient expectations for treatment and ultimately positively impacting their health-related quality of life (12). Patients often experience stress and anxiety related to their medical conditions and treatment. Positive expectations of physicians can help alleviate some of this stress and anxiety, leading to a better overall patient experience and potentially improved clinical outcomes (13). Clinical studies demonstrated that physicians’ expectations can influence a patient’s physiological response to treatment (14). Positive expectations might lead to the release of endorphins and other neurochemicals, contributing to an enhanced healing response and reduced pain perception (15). Moreover, the treatment expectations of physicians also reflect the treatment intention, which is associated with attitude, past behavior, perceived behavioral control, and subjective norms, thus influencing therapeutic modalities in clinical settings (16).

The current measures for assessing treatment expectations among clinicians in general hospitals remain insufficient. Many survey questionnaires have been developed for specific treatments or diseases, such as brain metastases in cancer (17), high tibial osteotomy for osteoarthritis (18), and idiopathic pulmonary fibrosis (19). Furthermore, the absence of a quantitative scale makes it challenging to quantitatively evaluate the impact of physicians’ treatment expectations on overall patient management and hinders comparisons across different hospital departments. Consequently, there is a compelling need to establish a quantitative tool for evaluating physicians’ treatment expectations that can be applied across various clinical departments. Therefore, we created the hospitalized patients’ expectations for treatment scale-clinician version (HOPE-C) to enhance our understanding of physicians’ treatment expectations and improve patient safety management systems.

Our study sought to validate a structured assessment tool to measure clinicians’ expectations for treatment. The primary objective was to provide a dependable and practical assessment instrument for forthcoming clinical applications, addressing the increasing demand for personalized healthcare and reinforcing a robust patient safety framework. In a prior phase of our research, we effectively validated the hospitalized patients’ expectations for treatment scale-patient version (HOPE-P), affirming its validity and reliability in assessing treatment expectations among hospitalized patients (20). In the present study, we leveraged a similar scale structure to that of HOPE-P to create an assessment tool focused on clinicians’ expectations rooted in their perspective on clinical treatment. Our aim is to establish a comprehensive assessment system for the expectations of patients and clinicians for treatment in future clinical practice.

## 2 Materials and methods

### 2.1 Formulation of the scale

In this study, we developed the HOPE-C, building upon the prior scale developed for patients (HOPE-P). Thirty-five experts representing various medical fields, including psychiatry, surgery, internal medicine, nursing, and medical management, collaborated in the development of this tool according to the inclusion criteria: (1) employment in general hospitals or psychiatric specialty hospitals within China; (2) specialization in fields such as clinical medicine, healthcare management, medical informatics, or nursing; (3) active engagement in doctor-patient relationship dynamics and patient safety management within their respective work and research areas. Additionally, we conducted interviews with 10 randomly selected inpatients at the Peking Union Medical College Hospital, covering a range of medical specialties, such as general surgery, orthopedics, vascular surgery, plastic surgery, gastroenterology, infectious medicine, and neurology. To refine the scale, we employed the Delphi method, gathering insights from both medical experts and patients. The question was, “What do patients expect from hospitalization and doctors? What do patients concern most?” After three rounds of extensive discussions, participants’ responses converged on three domains: how a patient is treated on a person-to-person level by the doctor (doctor-patient communication), whether the clinical condition will improve during hospitalization (treatment outcomes), and condition as a long-term problem (perceptions of long-term disease management). We designed the HOPE-C in alliance with HOPE-P to make it convenient for clinical usage; hence, clinicians could easily identify misunderstandings between doctors and patients.

### 2.2 Participants

A total of 200 patients, spanning five different departments, were enrolled from the Peking Union Medical College Hospital in China between March 2023 and September 2023. Fifty-one clinicians were randomly assigned to manage the enrolled patients. To be eligible, participants must be aged ≥ 10 years, hospitalized for > 24 h, anticipate discharge within a week, and demonstrate the ability to understand and cooperate with the study requirements. Exclusion criteria encompassed acute suicidal tendencies, limited writing proficiency, language barriers, organic brain disorders, cognitive impairment, dementia, and psychosis. All participants provided informed consent, indicating their understanding of the study’s procedures by signing the consent form. For participants aged < 18 years, supplementary informed consent was secured from their parents or legal guardians.

### 2.3 Administration

The HOPE-C scale was administered to clinicians within 24 h of their patients’ admission to the ward. This process was facilitated through a smartphone applet developed and supported by the Department of Psychological Medicine at Peking Union Medical College Hospital, which served as the platform for completing the HOPE-C scale. Specially trained investigators provided information about the research to patients and their assigned clinicians. They also supplied a QR code for the applet. Patients who consented to participate scanned the QR code using their mobile phones, accessed the main interface, and signed an informed consent form. For patients aged < 18 years, their parents or guardians authorized and signed additional informed consent forms.

Following the applet’s instructions and with the investigator’s assistance, the assigned clinicians provided essential information and completed the HOPE-C scale. The sociodemographic questionnaire collected data on age, gender, residence, marital status, family income, education level, employment status, and other pertinent details. Subsequently, 20 clinicians participated in a retest conducted 7 days after the initial completion of the HOPE-C scale to evaluate the test-retest reliability and the stability of the measurement tool over time.

### 2.4 Sample size evaluation

The sample size calculation was guided by “rules of thumb” or “blue chips.” These guidelines suggest that a minimal sample size, typically exceeding 200, is necessary to ensure sufficient statistical power for data analysis. Furthermore, these rules of thumb propose that the ratio of the number of individuals (*N*) to the number of measured variables (p) should ideally fall within the range of 5–10, with a minimum requirement of *N* > 100. A widely accepted standard is to have 10 cases per indicator variable. Considering these factors, the total sample size was determined to be 200, and the Kaiser-Meyer-Olkin (KMO) was used to assess the adequacy of the sampling.

### 2.5 Statistical analysis

Data analysis was performed using IBM SPSS version 28.0 and AMOS version 26.0. The significance level was set at a two-tailed *p*-value < 0.05 for the entire analysis.

#### 2.5.1 Descriptive statistics

Continuous variables were presented as median (25 and 75th percentiles), and categorical variables were represented as the count along with the corresponding percentage (*n* [%]). Kolmogorov–Smirnov test was applied to evaluate the normality of the data. Additionally, differences among groups in HOPE-C scores were assessed by the Mann–Whitney U test for 2-group comparison and the Kruskal–Wallis H test with Bonferroni adjustment for multiple-group comparison.

#### 2.5.2 Item analysis

The HOPE-C total score was divided into two groups based on high and low scores, with the upper 27% and lower 27% of scores forming the respective groups. A *t*-test was conducted to compare these groups, leading to the calculation of critical ratio (CR) values. We calculated the corrected associations between items and the total score to assess the strength of correlations between each item and the overall scale score. If the item-total correlation coefficient exceeded 0.4, it was considered a satisfactory result (21).

#### 2.5.3 Structural validity

We conducted an exploratory factor analysis (EFA) using IBM SPSS version 28.0 on a randomly selected half of the collected dataset to explore the previously unexamined structure of the HOPE-C scale. Before proceeding, we performed checks on the data suitability and sampling adequacy, which included the KMO measure and Bartlett’s test of sphericity. KMO > 0.70 and *p* < 0.001 for Bartlett’s test indicated adequate data suitability and sampling adequacy, signifying a substantial correlation between the items suitable for structure detection. We applied varimax rotation and extracted factors with eigenvalues > 1. A total factor loading > 60% was considered satisfactory (22). A confirmatory factor analysis (CFA) was performed using AMOS version 26.0 on the other half of the sample. The following criteria determined global model fit appropriateness: a root mean square residual (RMR) value < 0.05, a root mean square error of approximation (RMSEA) value ≤ 0.10, along with comparative fit index (CFI), normed fit index, non-normed fit index, incremental fit index, Tucker-Lewis index (TLI), and goodness of fit index values > 0.9 (23).

We calculated Cronbach’s α coefficients and McDonald’s ω coefficients for both the complete scale and individual subscales to assess the internal consistency of the recently developed tool. Additionally, we provided 95% confidence intervals (95% CI). Internal consistency was considered high when Cronbach’s α coefficient or McDonald’s ω coefficient exceeded 0.7, while a value > 0.9 suggests redundancy (24). For evaluating the 7-day test-retest reliability, we determined the Pearson correlation coefficient or Spearman correlation coefficient based on the results of the Kolmogorov–Smirnov test between the initial test and the retest.

## 3 Results

### 3.1 Descriptive statistics

The study involved 200 patients, with an average age of 43.50 ± 14.34 years, and 122 (61.0%) of them were male (Table 1). Among the clinicians assigned to the enrolled patients, 51 completed the HOPE-C. The mean total score on the HOPE-C scale was 37.66 ± 4.31, out of a maximum possible score of 45. Subscale scores were as follows: 13.79 ± 1.78 for the doctor-patient communication expectations subscale (referred to as subscale A, covering items 1–3 with a maximal score of 15), 21.83 ± 2.92 for the clinicians’ treatment outcome–related expectations subscale (referred to as subscale B, encompassing items 4–8, with a maximal score of 25), and 2.06 ± 1.03 for the disease management expectancy subscale (subscale C, with a maximal score of 5). No statistically significant differences were detected in HOPE-C total scores and subscale scores based on patients’ gender, employment status, place of residence, monthly family income, or educational level.

**TABLE 1:** Demographic characteristics of the overall sample.

Variables	Number (%)	Overall Scale Mean ± SD	Subscale A Mean ± SD	Subscale B Mean ± SD	Subscale C Mean ± SD
Age	*N* = 200	*P* < 0.001*	*P* = 0.063	*P* < 0.001*	*P* = 0.822
10-20	2 (1.0%)	38.00 (35.00, -)	13.00 (11.00, -)	23.00 (21.00, -)	2.00 (1.00, -)
21-40	39 (19.5%)	41.00 (37.00, 42.00)	15.00 (14.00, 15.00)	24.00 (21.00, 25.00)	2.00 (1.00, 3.00)
41-60	79 (39.5%)	38.00 (36.00, 41.00)	15.00 (13.00, 15.00)	22.00 (20.00, 24.00)	2.00 (1.00, 2.00)
61-80	79 (39.5%)	37.00 (34.00, 40.00)	14.00 (12.00, 15.00)	20.00 (20.00, 23.00)	2.00 (2.00, 2.00)
>80	1 (0.5%)	35.00	12.00	21.00	2.00
Gender	*N* = 200	*P* = 0.062	*P* = 0.479	*P* = 0.085	*P* = 0.444
Male	122 (61.0%)	38.00 (34.00, 41.00)	15.00 (12.00, 15.00)	22.00 (20.00, 24.00)	2.00 (1.00, 2.00)
Female	78 (39.0%)	39.00 (36.00, 41.00)	15.00 (13.00, 15.00)	22.50 (20.00, 25.00)	2.00 (1.00, 3.00)
Residence	*N* = 200	*P* = 0.201	*P* = 0.639	*P* = 0.205	*P* = 0.959
Urban	175 (87.5%)	38.00 (35.00, 41.00)	15.00 (12.00, 15.00)	22.00 (20.00, 24.00)	2.00 (1.00, 2.00)
Rural	25 (12.5%)	39.00 (37.00, 41.00)	15.00 (14.00, 15.00)	22.00 (20.00, 25.00)	2.00 (1.00, 3.00)
Marital status	*N* = 200	*P* = 0.077	*P* = 0.081	*P* = 0.128	*P* = 0.293
Single	17 (8.5%)	40.00 (36.50, 41.50)	15.00 (12.50, 15.00)	23.00 (20.50, 25.00)	2.00 (1.00, 4.00)
Married	167 (83.5%)	38.00 (35.00, 41.00)	15.00 (12.00, 15.00)	22.00 (20.00, 24.00)	2.00 (1.00, 2.00)
Divorced	5 (2.5%)	39.00 (36.50, 40.50)	15.00 (13.50, 15.00)	22.00 (21.00, 24.50)	1.00 (1.00, 2.50)
Widowed	5 (2.5%)	35.00 (33.50, 36.00)	12.00 (12.00, 12.50)	20.00 (19.50, 21.00)	2.00 (2.00, 3.00)
Other	6 (3.0%)	39.00 (37.25, 41.50)	15.00 (12.50, 15.00)	23.00 (20.75, 25.00)	3.00 (1.00, 3.00)
Employment status	*N* = 200	*P* = 0.060	*P* = 0.666	*P* = 0.106	*P* = 0.744
Student	6 (3.0%)	40.50 (36.50, 43.25)	15.00 (11.00, 15.00)	25.00 (21.00, 25.00)	3.00 (1.00, 4.00)
Employed	74 (37.0%)	39.00 (36.00, 41.00)	15.00 (13.00, 15.00)	23.00 (20.00, 25.00)	2.00 (1.00, 2.25)
Unemployed	24 (12.0%)	38.00 (35.00, 41.00)	15.00 (12.00, 15.00)	22.00 (20.00, 24.75)	2.00 (1.00, 2.75)
Retired	84 (42.0%)	37.00 (34.00, 40.00)	14.00 (12.00, 15.00)	21.00 (20.00, 24.00)	2.00 (1.00, 2.00)
Other	12 (6.0%)	38.50 (37.00, 40.50)	15.00 (13.25, 15.00)	22.50 (21.25, 24.00)	2.00 (1.00, 2.75)
Educational level	*N* = 200	*P* = 0.698	*P* = 0.815	*P* = 0.566	*P* = 0.968
Elementary	12 (6.0%)	39.00 (37.25, 40.00)	14.50 (14.00, 15.00)	22.00 (20.25, 24.00)	2.00 (1.00,2.75)
Junior	21 (20.5%)	38.00 (34.50, 41.00)	14.00 (12.00, 15.00)	21.00 (20.00, 24.00)	2.00 (1.00, 3.00)
High school	41 (20.5%)	38.00 (34.50, 41.00)	15.00 (13.00, 15.00)	22.00 (20.00, 24.00)	2.00 (1.00, 2.00)
College or higher	106 (53.0%)	38.50 (35.00, 41.00)	15.00 (12.00, 15.00)	22.00 (20.00, 25.00)	2.00 (1.00, 2.00)
Monthly family income	*N* = 200	*P* = 0.780	*P* = 0.986	*P* = 0.828	*P* = 0.519
<4,000 RMB	41 (20.5%)	38.00 (35.00, 41.00)	15.00 (12.00, 15.00)	22.00 (20.00, 24.00)	2.00 (1.00, 3.00)
4,000-8,000 RMB	71 (35.5%)	38.00 (35.00, 41.00)	15.00 (12.00, 15.00)	21.00 (20.00, 25.00)	2.00 (1.00, 2.00)
>8,000 RMB	88 (44.0%)	39.00 (35.00, 41.00)	15.00 (12.00, 15.00)	22.00 (20.00, 24.00)	2.00 (1.00, 2.00)
Wards	*N* = 200	*P* < 0.001*	*P* < 0.001*	*P* < 0.001*	*P* < 0.001*
Orthopedics	31 (15.5%)	40.00 (39.00, 41.00)	15.00 (14.50, 15.00)	24.00 (22.00, 25.00)	2.00 (1.00, 3.00)
Urology	102 (51.0%)	36.00 (34.00, 39.00)	13.00 (12.00, 15.00)	20.00 (20.00, 22.00)	2.00 (2.00, 2.00)
General Surgery	26 (13.0%)	41.00 (39.00, 42.00)	15.00 (14.00, 15.00)	24.50 (23.00, 25.00)	2.00 (1.00, 3.00)
Cardiology	15 (7.5%)	40.00 (37.50, 41.00)	15.00 (14.50, 15.00)	24.00 (23.00, 25.00)	1.00 (1.00, 1.00)
Endocrinology	26 (13.0%)	38.50 (37.00, 40.00)	15.00 (15.00, 15.00)	22.00 (21.00, 23.00)	1.00 (1.00, 2.00)
Medical Condition					
Orthopedics	*N* = 31	*P* = 0.290	*P* = 0.515	*P* = 0.677	*P* = 0.879
Cervical Spondylosis	8 (25.8%)	40.00 (31.75, 41.00)	15.00 (15.00, 15.00)	23.00 (14.75, 25.00)	2.00 (1.00, 2.00)
Scoliosis	5 (16.1%)	41.00 (40.50, 43.00)	15.00 (14.50, 15.00)	25.00 (23.00, 25.00)	3.00 (1.00, 2.00)
Atlantoaxial Dislocation	4 (12.9%)	40.00 (36.25, 20.75)	15.00 (13.50, 15.00)	23.00 (20.75, 24.50)	2.00 (1.25, 2.00)
Lumbar Spinal Stenosis	11 (35.5%)	40.00 (39.00, 41.00)	15.00 (14.00, 15.00)	24.00 (22.00, 24.00)	2.00 (1.00,3.00)
Others	2 (9.7%)	41.00 (38.00, -)	15.00 (15.00, 15.00)	24.00 (20.00, -)	3.00 (1.00, -)
Urology	*N* = 102	*P* = 0.103	*P* = 0.291	*P* = 0.081	*P* = 0.459
Renal Tumor	24 (23.5%)	38.00 (35.00, 40.00)	14.50 (12.00, 15.00)	21.00 (20.00, 23.00)	2.00 (2.00,3.00)
Bladder Tumor	18 (17.6%)	34.50 (34.00, 38.25)	12.50 (12.00, 15.00)	20.00 (20.00, 20.50)	2.00 (2.00,2.25)
Prostatic Cancer	13 (12.7%)	34.00 (33.50, 36.50)	12.00 (12.00, 14.50)	20.00 (19.00, 20.50)	2.00 (1.50, 2.00)
Adrenal Tumors	27 (26.5%)	37.00 (34.00, 41.00)	13.00 (12.00, 15.00)	20.00 (20.00, 25.00)	2.00 (2.00, 3.00)
Urinary Caculus	10 (9.8%)	34.50 (34.00, 36.50)	12.00 (12.00, 13.25)	20.00 (20.00, 22.00)	2.00 (1.75, 2.50)
Others	10 (9.8%)	35.50 (33.00, 41.00)	13.00 (11.75, 15.00)	20.00 (19.75, 24.25)	2.00 (1.75, 4.00)
General Surgery	*N* = 26	*P* = 0.593	*P* = 0.252	*P* = 0.132	*P* = 0.486
Pancreatic Neoplasm	6 (23.1%)	40.00 (37.75, 43.00)	15.00 (14.00, 15.00)	22.50 (21.00, 24.25)	3.00 (1.75, 4.00)
Thyroid Nodule	4 (15.4%)	40.50 (36.25, 41.75)	14.00 (11.75, 14.75)	24.50 (21.75, 25.00)	2.00 (1.00, 3.75)
Thyroid Cancer	13 (50.0%)	41.00 (39.50, 41.50)	15.00 (14.00, 15.00)	25.00 (23.50, 25.00)	2.00 (1.00, 2.00)
Others	3 (11.5%)	42.00 (41.00, -)	15.00 (15.00, 15.00)	25.00 (25.00, 25.00)	2.00 (1.00, -)
Cardiology	*N* = 15	*P* = 0.863	*P* = 0.384	*P* = 0.776	*P* = 0.253
Coronary Atherosclerotic Heart Disease	7 (46.7%)	38.00 (37.00, 41.00)	15.00 (13.00, 15.00)	25.00 (23.00, 25.00)	1.00 (1.00, 1.00)
Atrial Fibrillation	4 (26.7%)	39.50 (39.00, 40.75)	15.00 (15.00, 15.00)	23.00 (23.00, 24.50)	1.00 (1.00, 1.75)
Others	4 (26.7%)	40.50 (16.75, 41.00)	15.00 (6.00, 15.00)	24.50 (9.75, 25.00)	1.00 (1.00, 1.00)
Endocrinology	*N* = 26	*P*- = 0.909	*P* = 0.599	*P* = 0.815	*P* = 0.468
Diabetes Mellitus	7 (26.9%)	38.00 (37.00, 40.00)	15.00 (15.00, 15.00)	21.00 (21.00, 24.00)	1.00 (1.00, 1.00)
Insulinoma	4 (15.4%)	38.50 (37.25, 39.75)	15.00 (15.00, 15.00)	21.50 (20.25, 22.75)	2.00 (1.25, 2.75)
Cushing Syndrome	3 (11.5%)	38.00 (38.00, -)	15.00 (15.00, 15.00)	22.00 (22.00, -)	1.00 (1.00, -)
Others	12 (46.2%)	39.00 (36.00, 40.50)	15.00 (14.25, 15.00)	22.50 (20.50, 23.00)	1.00 (1.00, 2.00)

Mann-Whitney U test was used for two group comparison and Kruskal-Wallis H test with Bonferroni adjustment for multiple group comparison.

*p < 0.01.

However, notable variations were observed in the total HOPE-C scores among patients of different age groups (*H* = 20.335, *p* < 0.001) and among patients in various hospital departments (*H* = 44.786, *p* < 0.001). Patients aged 21–40 years achieved the highest total HOPE-C scores (41.00 [37.00, 42.00]). Notably, clinicians in the urology department obtained lower scores compared to those in orthopedics (36.00 [34.00, 39.00] vs. 40.00 [39.00, 41.00], *p* < 0.001) and general surgery (36.00 [34.00, 39.00] vs. 41.00 [39.00, 42.00], *p* < 0.001). Concerning subscale-A scores, no significant differences were found among patients of different ages; however, clinicians in urology attained significantly lower scores compared to those in endocrinology (13.00 [12.00, 15.00] vs. 15.00 [15.00, 15.00], *p* < 0.001). Regarding subscale B, there were significant differences among patients of different ages (*H* = 21.483, *p* < 0.001), with those aged 21–40 years achieving the highest scores (24.00 [21.00, 25.00]). Clinicians in urology exhibited significantly lower scores compared to those in general surgery (20.00 [20.00, 22.00] vs. 24.50 [23.00, 25.00], *p* < 0.001). Furthermore, significant differences were observed among hospital departments in subscale C (*H* = 33.647, *p* < 0.001). Clinicians in the cardiology department expressed a greater expectation for long-term disease management compared to those in orthopedics, urology, and general surgery (1.00 [1.00, 1.00] vs. 2.00 [1.00, 3.00], *p* < 0.001; 1.00 [1.00, 1.00] vs. 2.00 [2.00, 2.00], *p* < 0.001; 1.00 [1.00, 1.00] vs. 2.00 [1.00, 3.00], *p* < 0.001). Considering medical conditions, 101 (50.5%) patients were diagnosed with cancer in different wards; however, there were no statistically significant differences in the HOPE-C score between distinct medical conditions.

### 3.2 Item analysis

The CR values for items 1–8 fell within the range of 8.528 to 13.598, and the distinctions between low- and high-scoring groups were all statistically significant. The scores for items 1–8 and the total score demonstrated significant correlations, with coefficients ranging from 0.744 to 0.927, all exceeding 0.40. Notably, item 9 had the lowest CR value (1.316) and displayed an insignificant correlation with the total score, indicating that this item fails to discern response variations among different investigators. This item lacked meaningful relevance in the survey and should be removed. The item analysis results are presented in Table 2.

**TABLE 2:** Item analysis.

	Item	Critical ration	Corrected item-total correlation
Subscale A: doctor-patient communication expectation	Q1. I listen to patient’s opinions on treatment	10.205**	0.830**
	Q2. During this hospitalization, I fully explains the state of illness and negotiates medical decision with patient	11.121**	0.879**
	Q3. During this hospitalization, I care my patient	10.499**	0.880**
Subscale B: treatment expectation	Q4. Through this hospitalization, the patient’s disease can be definitely diagnosed	11.417**	0.927**
	Q5. Through this hospitalization, the patient’s symptoms can be improved	10.294**	0.889**
	Q6. Through this hospitalization, the patient’s disease can be cured	8.528**	0.744**
	Q7. Through this hospitalization, the patient can restore work/family functions	13.598**	0.961**
	Q8. Through this hospitalization, the patient can take care of themselves	10.498**	0.893**
Subscale C: disease management expectancy by doctor	Q9. After this hospitalization, the patient need to mental long-term treatment	1.316	0.155

**p <0.01.

### 3.3 Structural validity analysis

Based on the item analysis results and the scale’s design concept, an EFA was conducted on a randomly selected half of the sample (*n* = 100) to ascertain the number of factors. According to the results of item analysis, EFA based on eight items conducted with the KMO statistics yielded a value of 0.875, with Bartlett’s test of sphericity showing χ^2^ = 617.936 (*p* < 0.001), indicating the suitability of the data for factor extraction. Subsequently, principal component analysis with varimax rotation was performed, identifying a single factor with eigenvalues exceeding 1. The single factor explained 65.551% of the total variance. Detailed item factor loadings are provided in Table 3.

**TABLE 3:** Results of exploratory factor analysis.

	Item	EFA (8 items)	EFA (9 items)		
		Loadings on factor	Loadings on factor 1	Loadings on factor 2	Loadings on factor 3
Subscale A: doctor-patient relationship expectation	Q1	0.681	0.742	−0.194	−0.210
	Q2	0.879	0.881	0.101	/
	Q3	0.871	0.850	0.164	−0.133
Subscale B: treatment expectation	Q4	0.851	0.842	0.178	0.140
	Q5	0.858	0.784	0.375	−0.118
	Q6	0.41		0.942	/
	Q7	0.912	0.885	0.228	/
	Q8	0.889	0.880	0.159	/
Subscale C: disease management expectancy by doctor	Q9	/	/	/	0.984

EFA, exploratory factor analysis.

Concerning the design concept and principle of this scale, we conducted EFA based on nine items to explore whether HOPE-C can be divided into three factors. The KMO statistics yielded a value of 0.866, with Bartlett’s test of sphericity demonstrating χ^2^ = 627.329 (*p* < 0.001). Three factors collectively explained 80.485% of the total variance. Upon thoroughly examining factor loadings, items 1–5 and 7–8 were grouped within one dimension, while items 6 and 9 were attributed to factors 2 and 3, respectively. As outlined in Supplementary Table 1, all items displayed factor loadings exceeding 0.6. However, the results of the EFA analysis using the 9-item scale did not align with the intended structure we designed for this scale.

**FIGURE 1:**
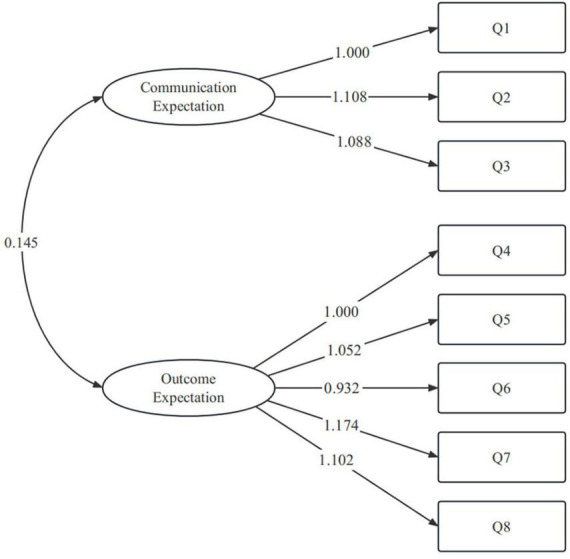
Factor structure of hospitalized patients’ expectations for treatment scale-clinician version (HOPE-C), based on an eight-item two-factor model.

Subsequently, a CFA using weighted least squares estimation was conducted on another randomly selected sample (*n* = 100) to assess the 2-factor model based on eight items, encompassing satisfaction with treatment experience– and outcome–related expectations. The outcomes revealed that the majority of items in the CFA model exhibited factor loadings surpassing 0.6 (Figure 1), and all model fit indices indicated a favorable fit (χ^2^/df = 1.237, RMSEA = 0.049, SRMR = 0.042, CFI = 0.989, TLI = 0.984).

### 3.4 Reliability analysis

An analysis of reliability using the 8-item 2-factor model demonstrated that the scale exhibited reliable performance. The McDonald’s ω coefficient for the complete HOPE-C scale was calculated at 0.883 (95% CI = [0.857, 0.906]). The McDonald’s ω coefficients for HOPE-C subscale A and subscale B were 0.820 (95% CI = [0.767, 0.856]) and 0.839 (95% CI = [0.802, 0.872]), respectively.

For the HOPE-C maximal scale, the Cronbach’s α coefficient was 0.884 (95% CI = [0.857, 0.906]). The Cronbach’s α coefficients for HOPE-C subscale A and subscale B were 0.816 (95% CI = [0.767, 0.856]) and 0.840 (95% CI = [0.802, 0.872]), respectively.

Furthermore, the test-retest reliability of the complete HOPE-C scale over a 7-day interval was 0.996 (*p* < 0.001). These analyses collectively indicated that the scale exhibits strong and reliable measurement properties.

## 4 Discussion

In this research, we enrolled 200 inpatients and 51 clinicians from a Chinese general hospital to investigate the reliability, validity, and psychometric characteristics of the HOPE-C scale. This scale was adapted from the HOPE-P and designed to assess clinicians’ multifaceted expectations for patients in a general hospital setting. Initially including three dimensions and nine items, the scale measures expectations related to the doctor-patient relationship, treatment outcomes, and disease management in Chinese healthcare.

Our findings indicated that the HOPE-C scale exhibits robust internal consistency, reliability, and validity. The results were consistent with a 2-factor model encompassing expectations tied to the treatment experience and satisfaction with treatment outcomes. This study confirmed the scale’s overall strong reliability and demonstrated satisfactory reliability and validity for the first two subscales. Thus, the HOPE-C scale allows a practical and accessible assessment of clinicians’ treatment expectations in Chinese healthcare culture.

The initial subscale of the HOPE-C evaluated expectations regarding doctor-patient communication and comprised three distinct items. Prior studies underscored variations in perceptions of doctor-patient communication between patients and clinicians (25). Consequently, Subscale A was tailored to measure doctor-patient communication expectations from the patient’s perspective. Effective doctor-patient communication is pivotal in nurturing patient trust in their healthcare providers, bolstering overall satisfaction, and indirectly influencing health outcomes, including symptom management and adherence to medical regimens. Thus, ensuring effective and efficient communication is a foundational component of strategies to deliver high-quality healthcare (26).

Within the HOPE-C scale, the three items that pertain to doctor-patient communication specifically address clinicians’ expectations regarding attentiveness to patient treatment opinions, transparent communication about the patient’s medical condition, active patient involvement in medical decision-making, and the demonstration of a compassionate demeanor. These items closely align with the core tenets of previous research on doctor-patient communication, which reflect the principles of patient autonomy, SDM, and a humanitarian approach—critical aspects of personalized medicine (27, 28). Our study results revealed distinctions in doctor-patient communication expectations among different hospital wards. Significantly, clinicians in the urology department expressed lower expectations regarding doctor-patient communication when compared to their counterparts in the orthopedics and endocrinology departments. This discrepancy suggests that perspectives on doctor-patient communication among clinicians still vary based on the clinical setting.

Extensive research on clinicians’ therapeutic expectations yielded compelling insights into the impact of physicians’ expectations regarding treatment outcomes on disease management results (29). In this study, outcome expectation pertains to a physician’s personal assessment of the potential benefits associated with a specific treatment plan that they intend to prescribe to a patient, considering the patient’s prognosis. Evaluating clinicians’ treatment outcome expectations is critical, as it serves as a guiding factor in clinical practice and aids in predicting treatment outcomes. Items 4–8 within the HOPE-C scale are specifically designed to assess physicians’ expectations concerning treatment outcomes. These items encompass various facets, including expectations for a clear diagnosis, disease improvement, recovery, and the restoration of functional capabilities. Additionally, patient age significantly influences physicians’ expectations regarding treatment outcomes. Furthermore, the nature of a patient’s specific medical condition can result in varying expectations regarding treatment outcomes and attitudes toward long-term disease management. Various treatment modalities and management approaches offered by clinicians, which are tailored to the unique characteristics of each medical condition, lead to diverse treatment experiences and outcomes. Consequently, this diversity affects the expectations of different clinicians in terms of treatment outcomes, aligning with the observations made in routine clinical practice. There is no apparent interaction between physicians’ treatment outcome–related expectations and doctor-patient communication. This suggests that doctor-patient communication does not influence the formulation of treatment expectations by physicians before they make clinical decisions. This finding underscores that, in contrast to patient expectations, physicians’ treatment outcome expectations are not substantially influenced by the quality of doctor-patient communication.

In line with our earlier investigation on the HOPE-P scale, this current study also does not support the inclusion of the disease management expectancy subscale within the HOPE-C. Several factors might contribute to this, including issues related to the item’s design or its standalone nature. According to our findings, we compared various CFA models and opted for an 8-item, 2-factor model for further analysis. Nevertheless, it remains crucial to focus on the concept of disease management expectancy for a holistic assessment of hospitalized patients’ treatment expectations, aiming to enhance personalized medical care. The CFA results revealed no correlation between individual items in the doctor-patient communication subscale and treatment-related outcome expectations, suggesting that clinicians’ expectations regarding treatment outcomes are independent of SDM. This finding implies that the SDM process might not be fully integrated into the routine clinical practice of inpatient care within a general hospital setting in China. In future research, exploration of the disparities in treatment expectations between patients and clinicians is required. Furthermore, examining whether and how differences in treatment expectations between patients and doctors impact clinical practice outcomes is essential.

Nonetheless, there were several limitations in this study. The inclusion of inpatients was restricted to a relatively small selection of departments, primarily encompassing three surgical and two internal medicine departments. Future research should consider enlarging the sample size and containing a wider array of departments to improve generalizability. Furthermore, while the current set of items in the HOPE-C scale aims to encompass critical dimensions, there is a need for further investigation to identify potential dimensions that warrant attention, thereby enhancing the scale’s comprehensiveness.

## 5 Conclusion

The HOPE-C scale demonstrates robust internal consistency, reliability, and validity, consistently aligning with the 2-factor satisfaction model. These two factors encompass doctor-patient communication and treatment outcome–related expectations, strengthening the scale’s reliability and relevance for assessing clinicians’ multifaceted treatment expectations.

## Data availability statement

The raw data supporting the conclusions of this article will be made available by the authors, without undue reservation.

## Ethics statement

The studies involving humans were approved by the Ethics Committee of Peking Union Medical College Hospital. The studies were conducted in accordance with the local legislation and institutional requirements. Written informed consent for participation in this study was provided by the participants’ legal guardians/next of kin.

## Author contributions

BD: Conceptualization, Data curation, Formal analysis, Writing – original draft. CX: Project administration, Writing – original draft. YW: Data curation, Writing – review & editing. TL: Resources, Writing – review & editing. YD: Resources, Writing – review & editing. YJ: Resources, Writing – review & editing. LS: Resources, Supervision, Writing – review & editing. XH: Resources, Writing – review & editing. WG: Resources, Writing – review & editing. JH: Project administration, Writing – review & editing. JC: Conceptualization, Supervision, Writing – review & editing. JW: Conceptualization, Funding acquisition, Supervision, Writing – review & editing.
